# Improving pregnant women’s diet and physical activity behaviours: the emergent role of health identity

**DOI:** 10.1186/s12884-020-02913-z

**Published:** 2020-04-25

**Authors:** T. Morris, S. Strömmer, C. Vogel, N. C. Harvey, C. Cooper, H. Inskip, K. Woods-Townsend, J. Baird, M. Barker, W. Lawrence

**Affiliations:** 1 MRC Lifecourse Epidemiology Unit, University of Southampton, Southampton General Hospital, Southampton, SO16 6YD UK; 2 NIHR Southampton Biomedical Research Centre, University of Southampton and University Hospital Southampton NHS Foundation Trust, Southampton, UK; 3 Southampton Education School, Faculty of Social and Human Sciences, University of Southampton, Southampton, UK

**Keywords:** Pregnancy, Diet, Physical activity, Health behaviour, Behaviour change, Identity, Intervention, Thematic analysis

## Abstract

**Background:**

Women who gain too much weight in pregnancy are at increased risk of disease and of having children with increased risk. Interventions to improve health behaviours are usually designed for a general population of pregnant women, and trial outcomes show an average impact that does not represent the differences between individuals. To inform the development of future interventions, this study explored the factors that influenced women’s diet and physical activity during pregnancy and aimed to identify the needs of these women with regards to lifestyle support.

**Methods:**

Women who completed a trial of vitamin D supplementation and nurse support in pregnancy were invited to take part in an interview. Seventeen women were interviewed about their lifestyles during pregnancy, the support they had, and the support they wanted. Interview transcripts were coded thematically and analysed to understand the factors that influenced the diets and physical activity levels of these women and their engagement with resources that could provide support.

**Results:**

Women identified barriers to eating well or being physically active, and pregnancy-specific issues like nausea and pain were common. Women’s interest in maintaining a healthy lifestyle and their engagement with lifestyle support was related to the extent to which they self-identified as healthy people. Health-disengaged women were disinterested in talking about their lifestyles while health-focused women did not feel that they needed extra support. Women between these ends of the ‘health identity’ spectrum were interested in improving their health, and were able to identify barriers as well as sources of support.

**Conclusions:**

Lifestyle interventions in pregnancy should be adapted to meet the needs of individuals with different health identities, and encouraging a change in health identity may be one way of supporting sustained change in health behaviours.

## Background

A woman’s health behaviours during pregnancy have lifelong implications for her own health and the health of her child. Studies have linked obesity and excessive gestational weight gain (GWG) with adverse pregnancy outcomes for the mother including gestational diabetes [1, 2], pre-eclampsia [3, 4] and an increased risk of developing type 2 diabetes [5, 6]. Offspring of these women are also at increased risk of overweight and obesity in childhood and of type 2 diabetes in adulthood [7, 8]. While there are no official guidelines for GWG in the UK, nearly half of women gain weight that exceeds United States Institute of Medicine guidelines during pregnancy [9].

Having a healthy diet and being sufficiently active during pregnancy can help women gain weight within the guidelines, potentially reducing the risk of the adverse outcomes described above [10]. Beyond any impact on GWG, diet and physical activity during pregnancy have been shown to affect maternal and infant outcomes. An unbalanced diet that does not meet a woman’s nutritional requirements in pregnancy has been associated with higher blood pressure in adult offspring [11] and is adversely associated with markers of metabolic health [12]. Similarly, being active during pregnancy has been shown to significantly reduce the odds of developing gestational diabetes [13, 14].

Despite the importance of maintaining a healthy lifestyle during pregnancy, most women do not meet established guidelines for dietary intake or physical activity [15–17]. Many interventions have been developed in response to this problem, but trial results often show non-significant, small or short-term effects [18–21]. Such studies tend to report considerable variation in participant engagement and this has consequences for participant outcomes. Those who engage with the intervention may experience benefits, such as lower GWG, compared with those who do not engage [18]. These findings suggest that the lack of engagement of some participants may help explain the small or non-significant average intervention effects that have been observed in so many trials. Appropriately tailoring interventions to meet individuals’ needs and preferences may improve engagement. However, understanding of why some people engage more than others with lifestyle interventions is limited [22].

Pregnancy is a unique phase when women may feel more motivated to improve their diets and physical activity levels and/or experience new barriers to changing behaviour, but there is limited understanding of factors that influence diet and physical activity in pregnancy [23, 24]. To develop a scalable intervention that can support women to adopt a healthy lifestyle during pregnancy, it is necessary to understand not only the key factors that influence women’s lifestyles during this period, but also why some women may engage more than others with an intervention. In order to fill these gaps in knowledge, a qualitative study with recently pregnant women who had participated in a trial evaluating a behaviour change intervention was undertaken.

The research questions for this study were:

1. What factors influence women’s diet and physical activity behaviours during pregnancy?
2. Why do some women engage more than others with interventions to improve diet and increase physical activity during pregnancy?

## Method

### Study design

For this qualitative study, semi-structured interviews were conducted with women who had taken part in the intervention arm of Southampton PRegnancy Intervention for the Next Generation (SPRING), an ongoing randomised controlled trial whose participants are pregnant women planning to give birth at the maternity hospital in Southampton, UK [25]. The trial registration number for SPRING is ISRCTN07227232 and ethics approval for this interview study was received from the South Central – Hampshire B Research Ethics Committee.

Using a 2^2^ factorial design, SPRING is a trial of the effects of i) vitamin D supplementation in pregnancy on the bone health of babies, and ii) behaviour change support from trained research nurses throughout pregnancy. Participants are randomised to vitamin D or placebo, and randomised again to receive Healthy Conversation Skills (HCS) behaviour change support or normal care. Women randomised to the HCS arm are seen by HCS-trained research nurses at each of the four study visits and receive one phone call from an HCS-trained nurse during the trial.

HCS has been described previously [26, 27]. Briefly, HCS training equips healthcare practitioners with skills to support patients to reflect on their health behaviours and empower them to find their own solutions to overcome barriers to change. This is facilitated through conversations that are driven by open-discovery questions, beginning with ‘how’ or ‘what,’ encouraging patients to reflect on the changes they would like to make, and on their personal circumstances, so they can plan their next steps. HCS-trained practitioners listen rather than give advice, and facilitate the setting of Specific, Measurable, Action-oriented, Realistic, Timed, Evaluated and Reviewed (SMARTER) plans.

### Participants

Women in the HCS intervention arm who completed SPRING between February and December 2016 were invited to take part in this interview study. They were sent a letter and an information sheet, describing the aims of the research, within 6 months of their final SPRING visit. Invitations were followed by phone calls and/or text messages to maximise participation. All women who expressed an interest by email or phone were interviewed. Invitation letters were sent out in batches to participants until enough interviews had been conducted to reach saturation [28]; this was when the interviewer and observer agreed that interviews were not yielding any new information. A total of 17 interviews were conducted.

### Interview process

Semi-structured interviews were employed to allow for a pre-determined set of topics to be discussed in an open and conversational way [29]. These were structured according to a discussion guide (see Table 1), and were audio-recorded. Face-to-face interviews in each woman’s home were conducted by female researchers following a semi-structured discussion guide. This guide was designed to explore women’s experiences with regard to their pregnancies, health behaviours, and sources of support. All interviews were conducted by the same researcher (TM), and one of two observers (WL for the first two and SS the other 15). The role of the observer was to listen to the interview, following along with the discussion guide, and ask any additional questions on topics that were felt not to be adequately addressed by the interviewer. Interviews lasted approximately 30 min.

**Table 1:** Interview discussion guide

**General health in pregnancy**	
What does it mean to you to have a healthy pregnancy? What factors are important?	
When you were pregnant, how did your health behaviours change?	
How important do you think it is to eat a healthy diet during pregnancy?	
How important do you think it is to exercise when you are pregnant?	
**Experience with behaviour change support during pregnancy**	
What kinds of things did you talk to the research nurses about during your appointments?	
What goals did you set with them?	
What were your reasons for setting those goals?	
How important was it to reach the goals you set?	
What factors in your life supported you to reach your goals? What made it difficult?	
How did your conversations with the nurses affect your health behaviours?	
What else do you think could have helped you reach your goals or have a healthier pregnancy?	

### Thematic analysis

Interviews were transcribed verbatim and analysed thematically using NVivo (QSR International) software. This process was done inductively, following guidelines set out by Braun and Clarke [30]. Initial codes were developed by creating ‘nodes’ in NVivo as new topics arose in the transcripts. Where a section of text fitted into more than one code, it was categorised under all appropriate codes. All transcripts were coded this way by TM, after which an initial coding frame was developed using a constant comparative approach in line with a relativist ontological and subjective epistemic position [31, 32]. Rigour was ensured by the double coding of a sample of the transcripts (6/17) by two researchers experienced in qualitative research and analysis (WL and CV). After the process of coding and double-coding was complete, the research team met to discuss the themes and determined how well they represented the data and how best to name and organise the themes into a final coding frame. The research team met regularly to discuss the coding frame and interpretation of findings to address the research questions.

## Results

### Participants

Seventeen women were interviewed and their demographic characteristics are shown in Table 2. The average age at interview was 33 years (ranging from 23 to 40 years). All participants had completed A-levels and most (76%) were educated to degree level or higher. Approximately half of the participants (47%) had only one child. More than half (53%) lived in the most affluent or second most affluent neighbourhoods in England according to Index of Multiple Deprivation (IMD) quintiles [33]. However, the range of deprivation from most deprived to least deprived was represented. Participation in SPRING was not restricted to women with a particular BMI and data about weight or weight gain were not collected as part of this interview study. Therefore, the findings presented herein do not represent a specific group with regard to body composition.

**Table 2:** Participant demographic characteristics

Description	Number (%)
Age at interview (y)	
21–25	1 (6%)
26–30	4 (24%)
31–35	5 (29%)
36–40	7 (41%)
Highest level of education	
A-level	4 (24%)
Higher national diploma	1 (6%)
Degree level or above	12 (71%)
Home index of multiple deprivation quintile	
1 – most deprived	1 (6%)
2	3 (18%)
3	4 (24%)
4	3 (18%)
5 – least deprived	6 (35%)
Number of children	
1	8 (47%)
2	8 (47%)
3	1 (6%)
Ethnicity	
White British	16 (94%)
East Asian	1 (6%)

### Key themes

Five themes relevant to the two research questions were identified in the data. These were: 1) What keeps me from improving my health? 2) What things in my life help me to be healthy? 3) How did I use pregnancy-specific resources? 4) How much did I engage with the research nurses’ support? and 5) Why do I want to be healthy? These themes are presented with their sub-themes in Table 3.

**Table 3:** Final coding frame

Theme	Description
1) What keeps me from improving my health?	Barriers to improving health or reaching goals
1a. The way pregnancy makes me feel	Pregnancy-specific experiences
1b. I’m still recovering from labour	Physical changes/impairments made it difficult to exercise post-birth
1c. My focus has changed since giving birth	Barriers related to having a new baby
1d. My health is not a priority	Lack of interest in own health
2) What things in my life help me to be healthy?	Facilitators to improving health or reaching goals
2a. I have always had a healthy lifestyle	Being healthy comes naturally
2b. People around me are healthy too	Social support for improving health behaviours
2c. My environment encourages healthy behaviours	Environmental factors such as food availability or exercise facilities
3) How did I use pregnancy-specific resources?	Use of available sources of information or support such as apps, books and classes
3a. Baby’s development	Reading about how the baby is growing and developing
3b. Specific concerns	Looking up specific symptoms or guidelines
3c. To help me improve my health	Advice or information related to improving diet or increasing physical activity
4) How much did I engage with the research nurses’ support?	Experiences and engagement with the HCS intervention
4a. I realised that they were trying to support me to set and reach health behaviour goals	Participants describe the HC skills that the nurses used
4b. The support I had helped me to be healthier	How the HCS intervention encouraged behaviour change
4c. I set goals and tried to meet them (or not)	Engagement with goal-setting and effort made to reach those goals
5) Why do I want to be healthy?	Motivators for wanting to improve diet or increase physical activity
5a. I want to do the best I can for my children	Focus on the baby’s health and development
5b. I want to stay healthy or get healthier	Focus on improvement or maintenance of own health
5c. I don’t want to be fat	Concerns about body weight

All themes are summarised below with illustrative quotes drawn from the interview transcripts. Each quote is followed by the woman’s age range at interview, number of children and home IMD quintile to show the spread of the data and to provide context.

#### What keeps me from improving my health?

For most women, pregnancy introduced new barriers to eating a balanced diet. While they believed diet was important, women often said that they chose foods that would help them manage morning sickness, and some talked about cravings. Starchy foods were often favoured in the first few months of pregnancy as they helped women cope with nausea.

*I hit six weeks and I just felt like absolute rubbish, and I pretty much lived on rice cakes and mashed potato for ages. It was just anything to settle my stomach, and that’s all I fancied. (26–30; 1 child; IMD 3)*

*I get hyperemesis and I vomit and vomit until about six months. Both times, until almost six months. And so I didn’t really eat anything except crisps and bread and Coke […] really starchy things to keep it down. (36–40; 2 children; IMD 3)*

Similarly, women found it difficult to remain physically active as they progressed through pregnancy.

*As I got more pregnant, it got harder. And he’s a very strong dog. He pulls a lot as well, so I got to the point where I was like, ‘I’m just not doing this anymore.’ I had quite a sore lower back in pregnancy as well. (26–30; 2 children; IMD 5)*

Fewer pregnancy-related barriers were discussed with regard to physical activity than diet. In contrast, the post-natal period appeared to influence physical activity levels more than pregnancy as women described being tired and needing to recover from childbirth.

*I had some problems with my episiotomy and the stitches came out. That was quite painful and sore, so I actually found walking quite difficult […] For the first two months I really couldn’t walk very far at all. (36–40; 1 child; IMD 2)*

For those who had more than one child, there were additional barriers such as lack of time or energy. This appeared to be true both during pregnancy and after giving birth.

*The second time you just sort of have to continue as you are because you’ve got someone else to think about. So actually, I didn’t make nearly as many changes to my life as I did the first time I was pregnant, when it was just me and my partner. (36–40; 2 children; IMD 3)*

*We haven’t been on as many walks as I used to do with [first child] when she was little. We used to go for a walk after my husband got home from work […] ‘Course we can’t do that now when we’ve got a toddler as well. (36–40; 2 children; IMD 3)*

Pregnancy-related barriers to improving diet and increasing physical activity were reported by most participants, regardless of their intention or desire to change these behaviours. However, one factor that was not necessarily related to pregnancy was a few women’s apparent lack of interest in their own health:

*I’m sure there’s plenty of other women out there that are a lot more together than me, and probably are out and, you know, jogging around the block at 7 o’clock in the morning or whatever. And you know, that’s just not me. (36–40; 2 children; IMD 3)*

Barriers to improving health behaviours were identified by most participants, and these were usually related to pregnancy or motherhood. On the other hand, some women were able to identify things in their lives that made it easier to be healthy.

#### What things in my life help me to be healthy?

Some participants seemed to find it easier than others to maintain a healthy lifestyle or reach their health behaviour goals during pregnancy. For a few women, staying healthy seemed to come naturally.

*I know how to eat healthily – I’ve always done it. (26–30; 1 child; IMD 4)*

*Oh, just that I have always been like, I really enjoy exercise. And then, you know… I didn’t want to not do anything and sort of stagnate when I was pregnant. I wanted to keep up doing something. So I think that was part of it too; the fact that I’m just naturally an active person. (31–35; 1 child; IMD 4)*

For those who said they were healthy people, this focus on health often extended to their households or social circles. Social support further enabled them to maintain healthy lifestyles during and post-pregnancy.

*I’m not panicking about any of it. As I said before, obviously with someone in the house who likes to cook, and he cycles a lot, so he wants to stay fit and healthy and the boys are always active so it’s quite easy. It’s not a household where everyone wants to eat you know, junk food. (31–35; 3 children; IMD 2)*

*My neighbour next door’s got a baby as well, so we’re quite close…You know the Couch to 5k?[…]I did that with her. That’s how I started running, and I eventually ran two 10k races after I had [my first child]… so we’re going to start that in a couple of weeks. (26–30; 2 children; IMD 5)*

In addition to their social networks, some women identified other factors in their environments that made it easier to maintain a healthy lifestyle. These included easy access to healthy food at home or work, and having responsibilities that kept them active.

*I was very lucky ‘cause where I work we have our lunches included… So it was really easy for me to eat quite healthily. I always had melon for my dessert instead of having the… oh I would allow myself the odd crumble here and there […] but the majority of the time, I would have sort of melon or strawberries or something like that. (31–35; 3 children; IMD 2)*

*I was really active because of [my son] and I didn’t want [the pregnancy] to affect him at all. Having a dog really helped because it meant that I would always go out and walk the dog. (31–35; 2 children; IMD 5)*

Those who were able to identify factors that helped them to stay healthy usually talked about long-term circumstances, which were not necessarily related to being pregnant, rather than available pregnancy-specific support.

#### How did I use pregnancy-specific resources?

Nearly all the women interviewed identified at least one resource that they used to find advice or information in pregnancy, including books, apps, classes, internet searches and social support groups. However, most of these resources were used for information about the development of the baby, or to look into a specific concern, rather than to support healthy behaviours:

*More interesting to see my baby’s the size of a honeydew melon this week rather than like, ‘you should be eating that honeydew melon.’ I guess I like to think I kind of know what it is to eat healthy. (31–35; 2 children; IMD 5)*

Tracking the development of the baby was mentioned frequently as an interesting feature, and was one of the primary uses for pregnancy apps.

*I always remember the Baby Centre ones because it was like, ‘this week your baby is the size of a tomato’ and that really grabbed me because I was like, I’d text my friends going, ‘banana.’ You know like tell them what size. (31–35; 2 children; IMD 5)*

Aside from the development of the baby, women were usually seeking information related to pregnancy-specific concerns or symptoms.

*I didn’t use anything as an ongoing thing, but if I had a query, you know, like, ‘oh, my hands have swollen,’ or whatever. So like**Babycentre.com**or something. (36–40; 2 children; IMD 3)*

*Many questions. Mainly many concerns about the development of my bump. Because there was an episode where I felt like my bump was developing not very fast. (31–35; 1 child; IMD 5)*

Only a few women used the internet to find diet-specific guidance:

*I did print a list of every food you’re supposed to eat, every vitamin you’re supposed to eat in pregnancy. So I tried to eat different things off that. (31–35; 2 children; IMD 5)*

About half of participants identified a resource they used to help them be physically active, including antenatal fitness classes and a goal-setting app. More than a third of participants did not specifically identify any resources for diet or physical activity support. Use of these resources appeared to differ between women who had had previous pregnancies and those who had been pregnant for the first time. In their first pregnancies, women tended to have more worries, and were more engaged with the pregnancy-specific information and resources available. Women who had been pregnant before seemed more relaxed about their pregnancies and appeared to have less interest in accessing information:

*When you’ve not been pregnant before, you think, ‘I mustn’t eat this sort of cheese, and I mustn’t eat this sort of fish, and I mustn’t drink and I mustn’t smoke and everything,’ and blah blah. And I don’t smoke and I don’t drink a lot anyway, but I think when you’ve done it, you sort of think, ‘actually…’ It’s not that it’s scare-mongering. It’s all very good advice, but I think the second time, you just have to be a bit sensible in yourself. (36–40; 2 children; IMD 3)*

Although most of the women interviewed did not specifically seek sources of diet or physical activity support, all of these women had five healthy conversations as part of SPRING, which aimed to support them to improve their health behaviours.

#### How much did I engage with the research nurses’ support?

SPRING participants were blinded to the HCS intervention, and were usually unaware that this was a component of the trial. Despite this, they were all able to talk about the way the research nurses asked them about their health and encouraged them to set goals. In general, women were positive about this support. Nearly all reported setting a physical activity goal and more than half of those also set a diet goal.

*I think actually the nurses were brilliant ‘cause they were never like, ‘well, you should be doing this…’ they weren’t telling me what to do. They were kind of asking me what I would like to do and getting me to think about what I want[…] It got me thinking and a bit more focused on what I should be eating and doing. (36–40; 2 children; IMD 3)*

There were two women who did not set any goals and one of these felt that the research nurse did not think she needed to make any changes.

*I didn’t really set any goals with them actually. We did talk about doing swimming […] but when she took my measurements she wasn’t overly concerned that I was putting on excess weight. So for her I think she said, ‘whatever you are doing is working, so just continue to do that.’ (36–40; 2 children; IMD 5)*

The other woman who did not set any goals had a more negative attitude toward her experience and repeatedly stated that she was not the kind of person to set goals:

*I’m just not that sort of person […] My goal was to just get through the pregnancy relatively well, so I didn’t want to set myself a goal of saying ‘I’m going to do yoga twice a week’ or whatever. Or ‘I’m not going to eat any chocolate or anything.’ I just kind of wanted to get through it. (36–40; 2 children; IMD 3)*

Women who set goals had a range of opinions about their helpfulness in supporting improved diet or increased physical activity. Some believed that they would have been just as healthy without the extra support, but most felt that having healthy conversations encouraged them to be healthier. Some women said that they appreciated the interest the research nurses showed in their health rather than only the development of the baby.

*It does make you keep up everything you set because you almost feel when you’ve got someone ringing you in a few months’ time, you want to be able to say to them, ‘yes, yes! I’ve still done it.’ (31–35; 3 children; IMD 2)*

*I wouldn’t necessarily say those goals were really important to me, but the general being fairly healthy […] it wasn’t so much about the goals, it was just the [trial] that got me thinking generally about what you’re eating, what you’re putting in your body. (31–35; 2 children; IMD 5)*

*It was quite nice to have that, that time to actually really think about* me *pregnant and not just the baby whilst I was pregnant, if that makes sense. (36–40; 2 children; IMD 3)*

There was considerable variation in engagement with the HCS intervention as some women had set clear goals towards which they were still working, whereas others had not set specific goals or could not remember them after their participation in SPRING had ended.

*I said to her that I was going to go back to Slimming World, which I have done. She asked me […] if I was still smoke-free. I said yeah. And basically, that was the major goal. (21–25; 2 children; IMD 1)*

*I don’t… I don’t know that I did, really. I found that quite hard. Yeah, they kept asking about things like that. I didn’t really set any goals*. *(36–40; 2 children; IMD 3)*

***I****: Do you think that talking about that with the nurses had any […] influence on your motivation to exercise, or to make sure you went and did it?*

***P****: Um… don’t think so. I mean they were lovely. Really lovely, really supportive, but I kind of knew I wanted to do that anyway. (26–30; 1 child; IMD 5)*

Participants engaged to varying degrees with the support offered by the research nurses. This variation appeared to be closely related to the priority women placed on their own health as well as the extent to which they felt they could benefit from this support. In addition, women differed in their reasons for wanting to set goals to improve their diets or be more physically active.

#### Why do I want to be healthy?

Of the factors women identified as motivating them to reach their goals or maintain a healthy lifestyle, the most common were: the desire to do the best they could for their child; concern for their own health; and the desire to lose weight after pregnancy or not gain too much weight during pregnancy. Only a few women were primarily motivated to eat a healthier diet because they believed that their baby would benefit.

*I do think that eating healthy is more important because that’s what they get, isn’t it, from you. (21–25; 2 children; IMD 1)*

The same woman was very focused on her children’s health, and stated that her children were her motivation for staying healthy.

*I don’t want to teach my children that they’ve got to be skinny because they don’t have to be skinny. They just have to be healthy, and that’s what I want. So I can’t teach them not to smoke if I’m smoking. I can’t teach them not to drink if I’m an alcoholic. I can’t teach them to be healthy if I’m not healthy myself. (21–25; 2 children; IMD 1)*

Most women, though, were also interested in their own health and how it would be affected by their pregnancies.

*I think to be honest it was probably for me rather than for the baby […] Just before I got pregnant I had been quite healthy because I had been training to do the half marathon […] I guess I just didn’t want to completely go back to where I was before I started. (31–35; 1 child; IMD 2)*

The focus was often related to weight gain and retention post-pregnancy.

*I just don’t want to be this fat mum waddling up to the school gates every day to pick up their children, you know? [...] But also, I’m worried about keeling over and having a heart attack because there’s been problems in my family – heart problems[…] I’m overweight and I don’t really look after myself and I’ve got two children. Something like that could happen to me, and I want to be healthy. (26–30; 2 children; IMD 5)*

*I think some people can slip into, ‘oh, I’m fat now.’ Then if you have another pregnancy… you know, I was quite scared about keeping my weight then having another baby and then just magnituding* [sic]. *I’ve seen a lot of people do that; they kind of put on four stone and they were healthy people, you know? (31–35; 2 children; IMD 5)*

**Fig. 1:**
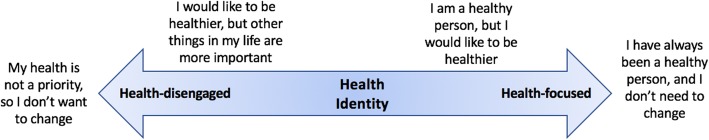
The proposed spectrum of health identity extends from ‘health-disengaged to ‘health-focused’

For some, the concern about health and weight was about not wanting to become an unhealthy person as this was not who they had been before having children. For others who had previously struggled to maintain a healthy weight or lifestyle, concerns about the effects of pregnancy reflected a general desire to be healthier. The extent to which women in this study viewed themselves as healthy can be conceptualised as ‘health identity’ where women’s perceptions of themselves fit on a spectrum that ranges from ‘health-disengaged’ to ‘health-focused’. Figure 1 is a visual representation of this concept and its relation to women’s openness to change. The statements that fall on different points in the figure are not quotes from the interviews, but were informed by this study and are intended to illustrate the range of responses.

## Discussion

This qualitative study aimed to address two research questions to inform the development of interventions for supporting pregnant women to improve their health behaviours. Firstly, we aimed to identify key factors that influenced diet and physical activity during pregnancy. Secondly, we explored women’s engagement with available lifestyle support in order to better understand why some women engage more than others.

In response to the first research question, this study identified pregnancy-specific factors that influenced diet and physical activity. Pregnancy can introduce new barriers such as nausea, pain and fatigue, making it a particularly difficult time to adopt healthier behaviours. Other qualitative studies have similarly found that physical symptoms introduced a barrier to physical activity and to engaging with a weight management intervention [34, 35]. There is, however, a paucity of observational data on pregnancy-related barriers to healthy eating. The current study also found that interest in the health and development of the fetus can provide motivation to make positive lifestyle changes, as can the desire to maintain a level of fitness and avoid gaining too much weight. This supports the idea that pregnancy can represent a ‘teachable moment’ for improving diet or increasing physical activity, which has been proposed by others [36].

The more novel finding of this study is in relation to the second research question. That is, women’s engagement with HCS and other sources of support appeared to be related to the priority they placed on their own health, and the extent to which they identified as healthy people. Indeed, the role of identity and its relation to health was an important factor that emerged from these data, and is summarised in the concept of ‘health identity’. Those whose health identity was always to have been healthy, or the kind of person to stay active, more often reported maintaining healthier lifestyles than those who did not identify as being particularly healthy.

Women’s health identities appeared to influence their engagement with available support and the idea of making a change. Women who did not prioritise their health were less likely to *want* to change their behaviours and did not really engage with the goal-setting element of the HCS intervention; nor did they seek lifestyle support through available services. At the other end of the spectrum, women who viewed themselves as healthy people and thus already prioritised their health, were unlikely to feel that they *needed* to set goals or change their behaviours. Those who fell between these two extremes were more likely to show interest in improving their health behaviours and to engage with available support. These women often had more to say about their goals in the interview and had made an effort to change their health behaviours.

The role of pregnancy in motivating women to change their health behaviours appeared also to vary along the health identity spectrum. For the most health-disengaged, pregnancy did not seem to provide significant motivation to set goals or change behaviour. For others who were relatively health-disengaged, diet and physical activity were not usually a major focus or priority, but pregnancy and motherhood provided extra motivation to make a change for their children’s benefit. Women who were near the middle of the spectrum identified as somewhat healthy people, and were not solely motivated by pregnancy or baby-related factors, but rather they were also interested in maintaining or improving their own health. Theme 5 suggests that the desire to be healthy was sometimes explicitly linked to women’s identities. The most health-focused women usually did not report making any significant changes to their behaviours because they felt they were already doing what they needed to stay healthy and have a healthy baby.

The relationship between health and identity has been described before [37–42]. Often, this work has focused on people with particular health conditions or disabilities and examined the effects of these conditions on a person’s identity [37]. However, some research has examined behaviour-related constructs including exercise identity [41] and healthy-eater identity [40]. These studies found that identity, in combination with self-efficacy, was an important determinant of health behaviours amongst university students. Similarly, a few studies have shown that exercise identity was associated with exercise adherence [41, 42]. While limited, the existing research on health behaviours and identity lends support to the idea that a woman’s health identity, in conjunction with other psychological factors, may predict her diet quality or level of physical activity. The current study extends this work by focusing on pregnancy, and by describing the role of health identity in influencing both women’s behaviours during this period and women’s engagement with health behaviour change interventions. Previous studies, though not explicitly referring to health identity, have shown that understanding the way women view themselves and their weight can help with personalising interventions to improve women’s engagement [43, 44].

### Strengths and limitations

The methods employed in this study were appropriate and effective for addressing the research questions. Participants recruited to the interviews had all been recently pregnant and were exposed to HCS, making them ideally placed to discuss both the factors that influenced their diet and physical activity in pregnancy and their engagement with a health behaviour change intervention. The semi-structured interview guide approach allowed for the collection of a rich and unique dataset, from which the concept of health identity emerged, and the thematic analysis was conducted in accordance with established guidelines [30], ensuring a rigorous and transparent process.

While all participants represented the target population, there was limited diversity in the sample with regard to demographic characteristics as all women lived in and around Southampton, most were educated to degree level and almost all were white British. Furthermore, there were considerably fewer women who fell toward the ‘health-disengaged’ end of the spectrum than the ‘health-focused’ end, which may limit the transferability of findings. While the range of socio-economic status by home IMD was represented, more than half of participants (9/17) lived in the two most affluent quintiles.

A potential limitation in any qualitative study is bias introduced by the assumptions and beliefs of the researcher [30, 31, 45]. In order to ameliorate this effect, the epistemological position informing this study was made clear from the outset and regular reflection on biases was part of the process. All interviews were conducted with a second researcher who took the role of an observer and who could ask additional questions. This helped to reduce the bias that may arise if a single person were conducting all of the interviews alone. Similarly, two members of the research team double-coded a selection of transcripts, and the coding frame was revisited and discussed to ensure consistency and accurate reflection of arising themes. Finally, in examining the different themes and synthesising the results of the study, five members of the research team (TM, SS, WL, CV and MB) met to discuss the interpretation presented here.

### Implications for practice

This study has shown that pregnancy is indeed a unique period where a number of factors influence women’s health behaviours. Firstly, it is clear that pregnancy and the post-natal period introduce physical barriers such as nausea, pain and fatigue, which make it more difficult to eat a balanced diet or be physically active. While pregnancy is sometimes viewed as a ‘teachable moment’ where women are inclined to improve their health behaviours [36, 46, 47], it is necessary to acknowledge that it is also a time when it can feel particularly difficult to change. Behaviour change interventions may be more effective if they help women to focus on changes they *can* make rather than changes that would be ideal. Encouraging women to reflect on their individual circumstances and come up with their own ideas about what they can change, as is done through having ‘healthy conversations’, could be a powerful intervention component [26].

The main finding of this study was the idea that women have different health identities and that this identity affects their level of engagement with behaviour change support. For health-disengaged women, goal-setting interventions may not be effective in supporting improvements in health behaviours, suggesting that alternative strategies are required for this group. In the present study, only a few women showed signs of health-disengagement, so further research with different and more diverse groups of pregnant women is needed to explore their priorities and to identify strategies that can encourage them to change.

Those who were somewhat health-disengaged did not necessarily prioritise their own health, but were often motivated by their pregnancies, and engaged with health advice for their babies’ benefit. For these women, pregnancy and the transition to motherhood provide a particularly valuable opportunity for health and social care practitioners to intervene and support improvements in health behaviours. However, changes that are motivated by pregnancy may only be temporary, as is often the case with smoking cessation [48]. Data presented in this paper suggest that there are two opportunities for more effective working with somewhat health-disengaged women: 1) there is potential to motivate women to improve their health behaviours by appealing to their desire to do the best they can for their baby and ensuring they are aware of the potential consequences of (not) changing their health behaviours [47]; and 2) improvements in health behaviours may be more permanent if they are linked to the woman’s identity as a healthier person.

Most of the participants in this study fell at the health-focused end of the health identity spectrum. For these participants, pregnancy was an opportunity for additional awareness of their own health and body composition, and the HCS support and goal-setting provided by the SPRING research nurses was acceptable and often supported women to strive for a healthier lifestyle. Again, when these women make changes during pregnancy, this may encourage a shift in health identity, making the change a permanent part of how they view themselves and their lifestyles.

Women who were very health-focused believed that they did not need to change their behaviours because they had always been healthy. For these women, pregnancy may not require a major change in lifestyle, so goal-setting interventions may not be necessary. However, having a baby introduced new barriers for some women, and while the most health-focused felt that they did not need to make a change during pregnancy, they may find it is more difficult to maintain their healthy lifestyle after giving birth. Therefore, it could be beneficial to encourage women to think about how they will overcome new barriers in the future and adjust to having a new baby or a growing family.

While the concept of health identity has not yet been incorporated into any diet or PA interventions, a recognisable example of using identity to facilitate behaviour change is in smoking cessation strategies. That is, a change in identity from ‘smoker’ to ‘ex-smoker’ or ‘non-smoker’ has been associated with successful quit attempts [49], while ‘smoker identity’ has been identified as a barrier to quitting [50]. It is possible that a similar principle could be applied to diet or physical activity change. If it is feasible to support a change in identity from ‘I am not someone who jogs’ to ‘I am an active person,’ or from health-disengaged to health-focused, such an intervention could lead to meaningful and sustained improvements in health behaviours.

As noted above, these conclusions are based on a relatively small and homogenous sample of women. Before developing new interventions, the concept of health identity should be investigated in other groups, using both qualitative and quantitative methods. This should include further exploration of health identity as a construct that influences health behaviours and openness to change, the potential impact of life events such as pregnancy on health identity, and development of a tool to assess health identity. Further work should then aim to develop and test methods of supporting women to move toward the health-focused end of the health identity spectrum, as well as identifying intervention components that are particularly effective for women with different health identities.

## Conclusions

Interventions that effectively support pregnant women to adopt healthier diets and increase their levels of PA have the potential to improve the women’s own health and the lifelong health of their children. If further investigation shows that health identity is related to lifestyle and engagement with lifestyle support among other groups of pregnant women, this concept may be a characteristic upon which intervention tailoring could be based. In order to operationalise this, a measure of health identity, which may or may not be pregnancy-specific, should be developed and validated. In addition, behaviour change techniques that support women to move along the health identity spectrum to be more health-focused should be developed and trialled as a means of supporting sustained, long-term change.

## Data Availability

Interview data cannot be made available as consent for sharing of complete transcripts was not obtained from participants.

## Abbreviations

GWG: Gestational weight gain
HCS: Healthy Conversation Skills
IMD: Index of multiple deprivation
SMARTER: Specific, Measurable, Action-oriented, Realistic, Timed, Evaluated and Reviewed
SPRING: Southampton PRegnancy Intervention for the Next Generation
